# The hibernating 100S complex is a target of ribosome-recycling factor and elongation factor G in *Staphylococcus aureus*

**DOI:** 10.1074/jbc.RA119.012307

**Published:** 2020-03-24

**Authors:** Arnab Basu, Kathryn E. Shields, Mee-Ngan F. Yap

**Affiliations:** ‡ Department of Biochemistry and Molecular Biology, Saint Louis University School of Medicine, Saint Louis, Missouri 63104; § Department of Microbiology-Immunology, Northwestern University Feinberg School of Medicine, Chicago, Illinois 60611

**Keywords:** ribosome, Staphylococcus aureus (S. aureus), GTPase, translation elongation factor, translation regulation, 100S ribosome, bacterial persistence, elongation factor G (EF-G), hibernation, ribosome-recycling factor (RRF)

## Abstract

The formation of translationally inactive 70S dimers (called 100S ribosomes) by hibernation-promoting factor is a widespread survival strategy among bacteria. Ribosome dimerization is thought to be reversible, with the dissociation of the 100S complexes enabling ribosome recycling for participation in new rounds of translation. The precise pathway of 100S ribosome recycling has been unclear. We previously found that the heat-shock GTPase HflX in the human pathogen *Staphylococcus aureus* is a minor disassembly factor. Cells lacking *hflX* do not accumulate 100S ribosomes unless they are subjected to heat exposure, suggesting the existence of an alternative pathway during nonstressed conditions. Here, we provide biochemical and genetic evidence that two essential translation factors, ribosome-recycling factor (RRF) and GTPase elongation factor G (EF-G), synergistically split 100S ribosomes in a GTP-dependent but tRNA translocation-independent manner. We found that although HflX and the RRF/EF-G pair are functionally interchangeable, HflX is expressed at low levels and is dispensable under normal growth conditions. The bacterial RRF/EF-G pair was previously known to target only the post-termination 70S complexes; our results reveal a new role in the reversal of ribosome hibernation that is intimately linked to bacterial pathogenesis, persister formation, stress responses, and ribosome integrity.

## Introduction

Bacterial ribosome hibernation has emerged as one of the pivotal cellular processes for entry into quiescence and subsequent resuscitation. Hibernation-promoting factor (HPF),^2^ either alone or in combination with ribosome modulation factor (RMF), induces the dimerization of 70S ribosomes to form the translational inactive 100S complexes in all bacterial phyla with the exception of Actinobacteria and hibernation factor YfiA (1, 2). The loss of HPF or RMF causes rapid ribosome degradation (3–5), translational derepression (4, 6, 7), reduced longevity (4, 8, 9), and antibiotic and stress susceptibility (10–12). *Staaphylococcus aureus* HPF is one of the predominant proteins induced upon host cell internalization and during infections (13, 14). For reviews of the topic, see Refs. 15–19.

Native 100S ribosomes from various bacteria are devoid of mRNA and tRNAs (20–24). In Firmicutes such as the human opportunistic pathogen *S. aureus*, an ∼200 amino acid-long HPF promotes ribosome dimerization via its self-dimerizing C-terminal domain, whereas its N-terminal domain occupies the tRNA- and mRNA-binding sites of the 30S subunits and thus sterically prevents translation (20–23). *S. aureus* 100S ribosomes (and HPF) are constitutively produced throughout the life cycle, as confirmed by time course immunoblotting and MS analyses of the HPF-bound 100S complexes (4, 25–27). A strong CodY-dependent promoter largely accounts for the high levels of HPF (28). The significance of 100S ribosomes during logarithmic growth is unclear, although they are thought to function as storage sites to preserve unused ribosomes (*e.g.* post-termination recycled ribosomes) from degradation (3–5, 29). In fact, 70S dimerization is strongly linked to the protection of ribosomes and the maintenance of active translation pools (3, 23, 30).

The hibernating 100S ribosomes serve as a reservoir to avoid futile translation and supply nutrient and translational machinery during bacterial regrowth from dormancy. To reactivate hibernating ribosomes for translation, 100S complexes need to be split into 70S monomers or 30S and 50S subunits concomitant with the removal of HPF. We previously showed that the evolutionarily conserved GTPase HflX is able to dissociate both 100S ribosomes and vacant 70S ribosomes in *S. aureus* but that GTP hydrolysis is required only for 100S complex splitting (31). *Escherichia coli* HflX rescues post-termination complex (PoTc)-like stalled 70S ribosomes from mRNA during thermal stress (32, 33). The expression levels of *hflX* in *S. aureus* and *E. coli* are undetectable during normal growth but are up-regulated by heat shock. The deletion of *hflX* only produces modest phenotypes (31, 32), implying that a more general housekeeping factor(s) is involved in the ribosome recycling of 100S complexes or stalled 70S complexes under nonstressed conditions. We posit that the alternative dissociation pathway of the 100S ribosomes involves a factor(s) that recognizes a PoTc-like substrate. The bacterial ribosome-recycling factor (RRF) and the GTPase elongation factor-G (EF-G) are known to break down the PoTc consisting of an mRNA and an uncharged P/E-site tRNA on a fully rotated 70S complex (34). The exact order of mRNA and tRNA release and 70S splitting remains controversial (35–39). In addition, PSRP1-induced hibernating ribosomes in chloroplasts, which are not dimerized and remain as 70S monomers, are bound with Chl-RRF at the intersubunit junction (40).

Here, we show that the 100S ribosome is a hitherto unknown target of RRF and EF-G in *S. aureus*. The splitting activity depends on GTP hydrolysis and not on the translocase activity of EF-G. Ectopic expression of HflX rescues the growth defect of an RRF-depleted strain, indicating functional redundancy of these factors. RRF/EF-G- and HflX-dependent pathways appear to functionally converge for the reactivation of hibernating ribosomes during stress adaptation.

## Results

### RRF and EF-G cooperatively dissociate the 100S ribosome in a GTP-dependent manner

**Figure 1.**
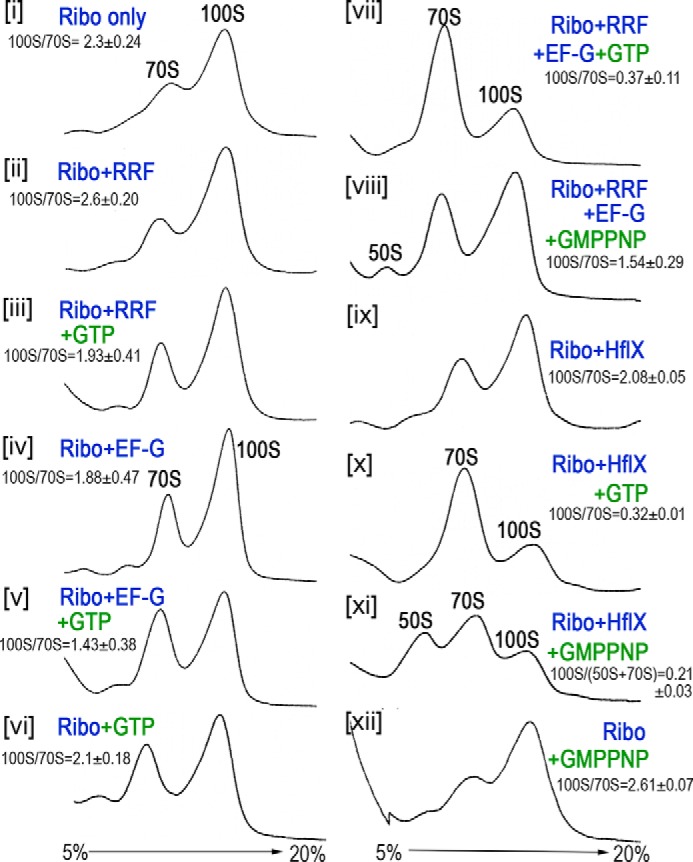
***In vitro* dissociation of the 100S ribosome by the RRF/EF-G pair and HflX in the presence and absence of guanosine analogs.** Reactions were programmed with 0.2 μm ribosomes, 2 μm proteins, and 2 mm GTP analogs and incubated at 37 °C for 30 min. The samples were centrifuged in a 5–20% sucrose gradient, and ribosome profiles were monitored from the absorbance at 254 nm (*y* axis). Quantification of the 100S to 70S ratios were obtained from three technical replicates (of two independently prepared ribosomes and recombinant proteins); mean ± S.D.

We performed *in vitro* 100S ribosome dissociation assays using purified recombinant RRF (encoded by *frr*) and EF-G (encoded by *fusA*) proteins in the presence and absence of GTP analogs. Native 100S ribosomes were isolated to ≥70% homogeneity from methicillin-resistant *S. aureus* (MRSA) USA300 through a two-step density gradient fractionation and ultracentrifugation protocol. The purified RRF, EF-G, and HflX proteins or GTP alone failed to dissociate the 100S complexes (Fig. 1, *panels i–v* and *ix*). More than 50% of the 100S ribosomes were converted into 70S monomers in the presence of RRF, EF-G, and GTP (*panel vii*) or HflX and GTP (*panel x*). The 70S ribosomes were not further dissociated by RRF/EF-G into 50S and 30S subunits because the reactions did not contain the anti-association factor IF3 (41). Some dissociation of 100S complexes were observed in reactions containing GTP plus either RRF or EF-G (*panels iii* and *v*). This is likely due to the residual activity of the ribosome-associated RRF or EF-G because high-salt wash that otherwise removes ribosome-bound factor(s), was omitted from our 100S ribosome preparation to prevent the release of HPF. When GTP was substituted with nonhydrolyzable guanosine 5′-(β,γ-imido)triphosphate (GMPPNP), which is a higher-affinity binder of ribosome-bound EF-G (42), the RRF/EF-G pair was unsuccessful in splitting the 100S complexes (*panel viii*). In contrast, GMPPNP induced the HflX-mediated dissociation of both the 100S and 70S complexes (*panel xi*), consistent with previous observations that GMPPNP is a more effective ligand of HflX in splitting the empty 70S ribosome (31, 32).

To validate these findings, we repeated the dissociation reactions using EF-G and RRF mutant proteins. Many loss-of-function mutations have been mapped to *E. coli* EF-G and RRF. For instance, a conserved Arg-29 is essential for GTP hydrolysis; a conserved Arg-59 affects tRNA translocation and ribosome binding but affects GTP hydrolysis only moderately; and H583K (His-572 in *S. aureus* numbering) conversion reduces tRNA translocation (43, 44). The substitution of Val-117 (V116 in *S. aureus* numbering) with an aspartate (*frr14* allele) in RRF renders a temperature-sensitivity phenotype (45).

**Figure 2.**
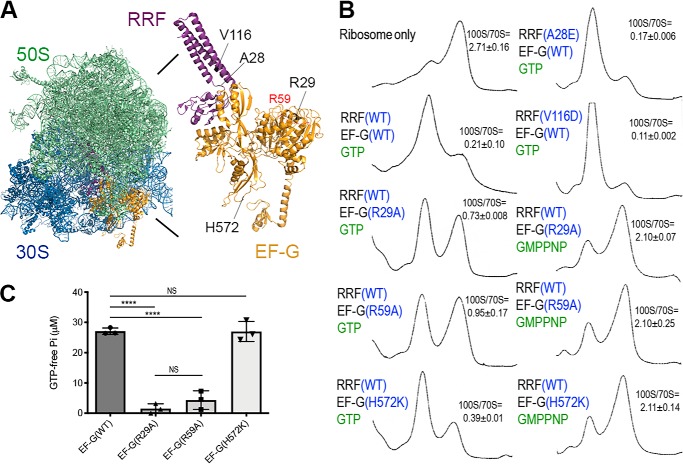
**The effects of RRF and EF-G mutations in 100S ribosome disassembly.** *A,* model of the RRF, EF-G and ribosome co-complex. A structural model was constructed from PDB accession numbers 4V54, 5OT7, and 4WPO. The mutations included in this study are indicated. R59 (*red*) was mapped to an unstructured region of EF-G in the crystal. *B,* the dissociation of 100S ribosomes is reduced in GTP-hydrolysis mutants (R29A and R59A) of EF-G but is unaffected by a mutation that compromises tRNA translocation (H572K). The dissociation reactions comprised 0.2 μm ribosomes, 2 μm proteins, and 2 mm GTP analogs and were incubated at 37 °C for 30 min. Ribosome profiles were analyzed via 5–20% sucrose density sedimentation, and the ribosomal species were monitored according to the absorbance at 254 nm. 100S to 70S ratios were obtained from three technical replicates (of two independently prepared ribosomes and recombinant proteins); mean ± S.D. *C,* malachite green GTPase assay showing the reduction of GTPase hydrolysis in the R29A and R59A mutants. The known translocation inactive H572K mutant does not present impaired GTPase activity. *Error bars* represent the S.E. obtained from three independent experiments using two different batches of purified proteins. *p* values were calculated by Student's unpaired *t* test, ****, *p* < 0.0001; *NS*, not significant.

We introduced mutations at the equivalent positions of *S. aureus* EF-G and RRF, in addition to a benign A28E mutation in a flexible region of RRF (Fig. 2*A*). The R29A and R59A mutants of EF-G are impaired in 100S ribosome dissociation, whereas the H572K mutant retains its splitting activity at the wildtype (WT) level (Fig. 2*B*). The dissociation of the 100S ribosome by the EF-G(H572K) is abolished upon GMPPNP substitution. The impairments caused by R29A and R59A are due in part to a decrease in GTP hydrolysis (Fig. 2*C*) and not to reduced ribosome binding (Figs. S1, *A* and *B*). Species-specific activities of RRF and EF-G have been observed between several bacterial homologs, and noncognate RRF and EF-G pairing is nonfunctional (46, 47). These variations could account for the difference in GTPase activity observed for *S. aureus* EF-G(R59A) compared with that of *E. coli* (43). We conclude that the translocation function (His-572) of EF-G is not required for 100S ribosome disassembly but that GTP hydrolysis is indispensable.

**Figure 3.**
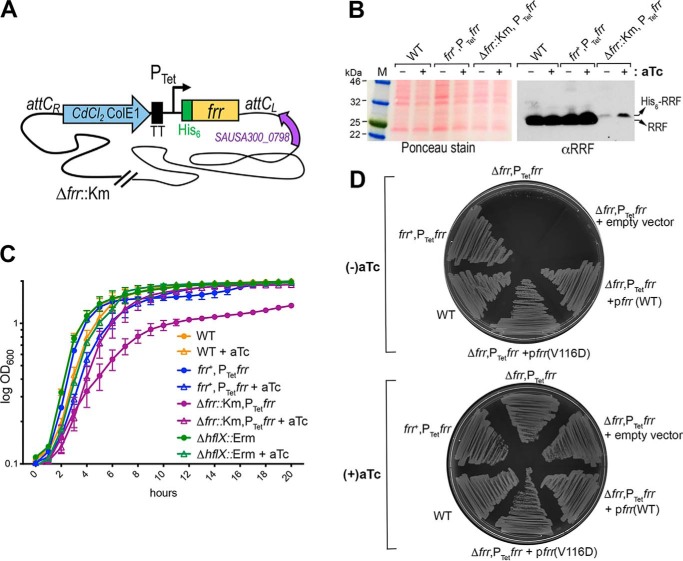
***In vivo* phenotypes of the RRF-depleted strain.** *A,* construction of the RRF-depleted strain. His_6_-tagged *frr* was first placed under the control of a tetracycline (*Tet*)-inducible promoter on a cadmium chloride (CdCl_2_)-resistant integration plasmid. The plasmid was integrated into the chromosomal *attC* site, resulting in an *S. aureus* strain carrying an inducible *frr* (P_Tet_*frr*) and a native copy of *frr* (*frr*^+^). The WT copy of *frr* was deleted (Δ*frr*::Km, confers kanamycin resistance) from the *frr*^+^,P_Tet_*frr* strain by homologous recombination in the presence of 400 ng/ml of aTc. *TT*, transcriptional terminator. *B,* relative expression levels of native RRF and aTc-inducible His_6_-RRF. The expression of RRF was analyzed by immunoblotting with anti-RRF (1:4,000 dilutions). RRF was expressed from the native locus at a much higher level than the His_6_-RRF. His_6_-RRF migrated slightly more slowly than the native RRF. *C,* growth defects of RRF depletion in liquid media. *S. aureus* cells were grown in TSB at 37 °C with and without aTc. Real-time cell density was monitored on a TECAN plate reader according to the absorbance at 600 nm. The addition of aTc slightly suppressed bacterial growth in all tested strains, but it restored the growth of the RRF-depleted strain to levels comparable with those in other strains with aTc supplementation. *Error bars* are S.E. from three independent experiments, and each experiment included five replicates. *D,* the growth defects of RRF depletion were more significant on solid agar plates. Single colonies of different *S. aureus* strains were streaked on Bacto^TM^ agar plates containing TSB base, and bacterial growth was recorded after 16 h of incubation at 37 °C. The RRF-depleted strain (Δ*frr*,P_Tet_*frr*) and its derivative carrying the empty pLI50 plasmid only formed a few microcolonies in the absence of aTc. WT *frr* and *frr*(V116D) expressed in the pLI50 plasmid fully rescued the growth defects of Δ*frr*,P_Tet_*frr*.

Contrary to our prediction, RRF(V116D) showed an increase in 100S ribosome dissociation, whereas RRF(A28E) was fully functional, as expected (Fig. 2*B*). Limited proteolysis showed that RRF(V116) protein was more accessible to chymotrypsin digestion and may therefore adopt different conformations than the WT RRF (Fig. S2*A*). In addition, RRF(V116D) bound to the ribosome and recruited EF-G as efficiently as WT RRF (Figs. S1, *C* and *D*, and S2, *B* and *C*). Consistent with these findings, the V116D mutant allele fully complemented the growth defect of an RRF-depleted strain (Fig. 3*D*).

### HflX complements RRF deficiency

RRF binds to the A-site of PoTc and recruits EF-G to initiate 70S ribosome splitting. RRF null is deleterious. EF-G is also an essential factor for the translocation step during elongation (34). To avoid complications arising from EF-G knockdown, we focused on the effects of RRF depletion on ribosome recycling.

To discern between endogenous RRF and exogenously introduced RRF, we constructed an RRF-depleted strain by deleting the *frr* (locus SAUSA300_1152) and placing a hexahistidine-tagged *frr* under the control of an anhydrotetracycline (aTc)-inducible promoter. The His_6_-*frr* allele was then integrated into a neutral site of the *S. aureus* chromosome (Fig. 3*A*). The same chromosomal integration system (48) has been widely used to complement *S. aureus* knockouts. The His-tagging strategy does not impair splitting activity of *S. aureus* RRF (Figs. 1 and 2*B*) nor compromises the efficiency of a reconstituted cell-free translation system containing *E. coli* His_6_-RRF (49). We found that the aTc-inducible promoter was leaky, allowing basal expression of His_6_-RRF without aTc and an upshift in the production of His_6_-RRF in the presence of an inducer (Fig. 3*B*). In contrast to the *hflX* knockout mutant, which lacked a phenotype at 37 °C but exhibited better heat tolerance at 47 °C (31), the depletion of RRF significantly reduced growth in liquid media; this defect was rescuable by the addition of aTc (Fig. 3*C*), confirming the direct role of RRF deficiency. The relatively low amount of His_6_-RRF (compared with the WT strain) was sufficient for growth (Fig. 3*B*), implying that the WT concentration of RRF is greater than that required for normal growth.

**Figure 4.**
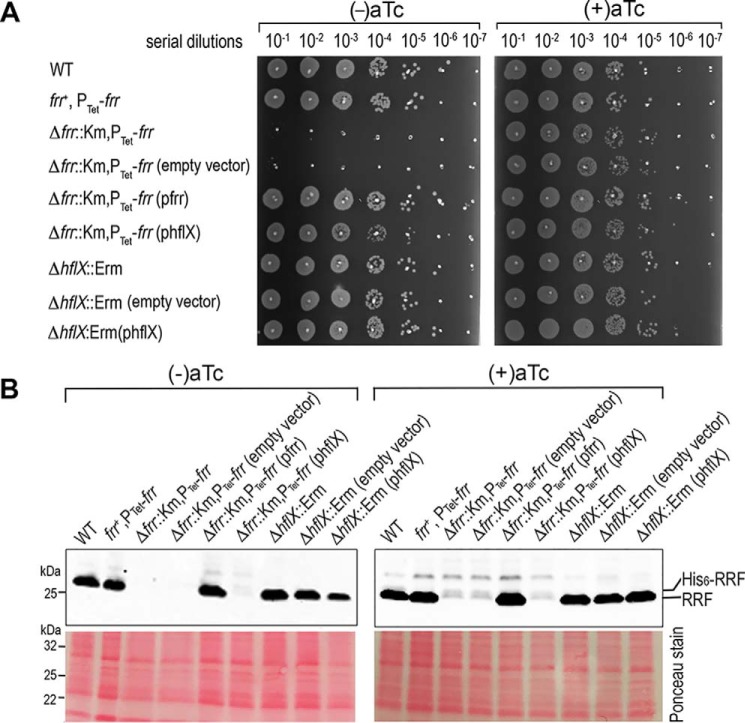
**HflX complements the deficiency of RRF.** *A,* spotting assay of the RRF-depleted strain and the *hflX* knockout harboring the indicated empty and complementation plasmids. Data are representative of three independent experiments. Cells were adjusted to an *A*_600_ of ∼0.2 and were serially diluted and spotted (3 μl/spot) on TSB-agar plates, then grown overnight at 37 °C to determine cell viability. The expression of *hflX* and *frr* in *trans* restored the growth of the RRF-depleted strain in the absence of anhydrotetracyline (aTc). *B,* expression of RRF in *S. aureus* TSB cultures with and without aTc. Western blots were performed using anti-RRF at 1:4,000 dilutions.

The growth defect of the RRF-depleted strain was accentuated on the solid agar media at all tested temperatures (30 °C-47 °C), in which only the supplementation of aTc supported growth (Fig. 3*D*). The severity of the growth defects observed on agar plates was only weakly associated with the type of gelling agents (Fig. S3). In the absence of aTc, RRF deficiency was fully complemented by expressing WT RRF or RRF(V116D) in a plasmid but not with an empty vector (Fig. 3*D*). Using the same complementation approach, we found that an *hflX* expressed in *trans* completely restored the growth defects of the RRF-depleted strain (Fig. 4*A*) and the expression of RRF in different genetic backgrounds was not affected by the introduction of plasmid (Fig. 4*B*). These results confirm that *hflX* functionally overlaps with RRF/EF-G in ribosome recycling. Furthermore, HflX is not expressed during growth at 37 °C and is exclusively up-regulated upon heat shock (Fig. S4) (31), explaining the dispensability of HflX under normal conditions.

### Consequences of inefficient ribosome recycling

**Figure 5.**
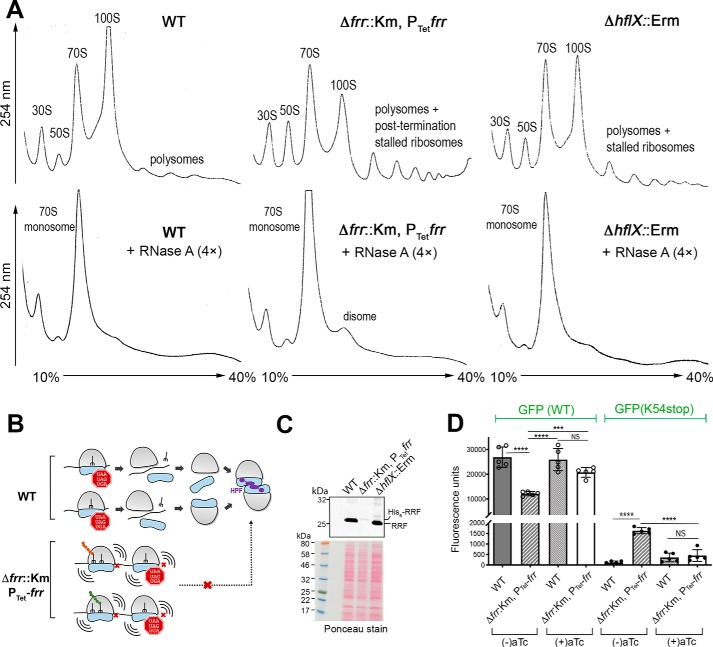
**Effects of RRF depletion on ribosome pools and stop codon read-through.** *A, top panels*, accumulation of unrecycled ribosomes and queuing of ribosomes in the RRF-depleted strain. Crude ribosomes were isolated from TSB cultures at *A*_600_∼ 1.4 (without the aTc inducer) grown at 37 °C and analyzed by 10–40% sucrose gradient sedimentation. No translational elongation inhibitor (*e.g.* chloramphenicol) was added throughout the experiments; thus, minimal polysomes were preserved in the WT strain. By contrast, unusually high levels of mixed polysomes and stalled ribosomes accumulated upon RRF depletion, which was also observed to a lesser extent in the *hflX* knockout mutant. *Lower panels*, conversion of polysomes into lower order ribosomal complexes by RNase A treatment. Excess RNase A (20 μg/ml) collapsed 100S ribosomes and the WT and Δ*hflX* polysomes, whereas a fraction of disome-like complexes in the RRF-depleted cells are resistant to RNase A. *B,* possible outcome of RRF depletion. RRF deficiency prevented the recycling of post-termination complexes, which in turn reduced the ribosome pools available for the formation of 100S ribosomes. *C,* analysis of RRF and His_6_-RRF production in the RRF-depleted strain and the *hflX* knockout mutant used in *A*. The deletion of *hflX* did not influence RRF expression. *D,* RRF deficiency reduced the translational capacity and promoted stop codon read-through. The fluorescence intensity of the GFP reporter plasmid (pDM4) was normalized according to the cell density and compared between the WT and RRF-depleted strains (Δ*frr*::Km,P_Tet_*frr*). RRF depletion reduced the expression of the WT GFP reporter. A stop codon was introduced at the Lys-54 position of the GFP to evaluate the degree of translational read-through. In the absence of the aTc inducer, ribosomes bypassed the Lys-54 stop codon, producing low levels of GFP. The addition of aTc to the Δ*frr*::Km,P_Tet_-*frr* strain suppressed read-through. *Error bars* are S.D. from five biological replicates. *p* values were calculated by Student's unpaired *t* test (*n* = 5, ***, *p* < 0.001; ****, *p* < 0.0001; *NS*, not significant).

We analyzed the effect of RRF depletion on ribosome pools by sucrose density gradient ultracentrifugation. Crude ribosomes were prepared from late log phase (an *A*_600_ of ∼1.4) cells grown without the aTc inducer. The cultures and sample preparation were free of any of the elongation inhibitors (*e.g.* chloramphenicol) that are normally used to preserve polysome species. Under these conditions, the WT cells accumulated 100S ribosomes but retained a very small fraction of polysomes (Fig. 5*A*, *top left*). In the RRF-depleted cells, PoTc could not be released from the mRNA templates, resulting in the queuing of the trailing ribosomes; these stalled polyribosomes were co-sedimented with the elongating polysomes (Fig. 5*A*, *top middle*). The lack of PoTc recycling reduced the level of ribosomal precursors available to form the 100S complexes, explaining the lower 100S peak in the RRF-depleted strain relative to the WT (Fig. 5*B*). The depletion of RRF was verified by immunoblotting (Fig. 5*C*). A very moderate effect on the 100S ribosome was observed in the *hflX* knockout (Fig. 5*A*, *top right*), consistent with previous findings (31, 32). We found that 5 μg/ml of RNase A was sufficient to fully collapse the *S. aureus* polysomes and 100S complexes but some polysomes in the RRF-depleted cells were resistant to ×4 access of RNase A and were preserved in a disome-like fraction (Fig. 5*A*, *bottom*), providing an additional clue to potential ribosome stacking at the termination codon.

To investigate the impact of RRF depletion on stop codon read-through, we compared the synthesis of green fluorescent protein (GFP) between the WT and the RRF-depleted strain carrying a pDM4 reporter plasmid (50). RRF depletion (no aTc) reduced WT GFP fluorescence by 3-fold, suggesting that the perturbation of ribosome recycling prevents new rounds of translation (Fig. 5*D*). When a termination codon was introduced at the Lys-54 position of *gfp*, RRF depletion promoted a low level of translational read-through, resulting in an increase in fluorescence intensity relative to the WT. The read-through was reversed upon the addition of aTc (Fig. 5*D*). We conclude that PoTc recycling and the formation of the 100S ribosome are interdependent and that RRF depletion reduces the translational capacity.

## Discussion

The recycling of PoTc, reactivation of the silent 100S ribosomes, and prevention of ribosome degradation are cost-saving activities because ribosome biogenesis is the most energy-consuming cellular process. A recent study offered the first clue that 100S dimers are disassembled and converted into active ribosomes in a reconstituted cell-free translation system consisting solely of purified components, including RRF and EF-G, but not the HflX (30).

**Figure 6.**
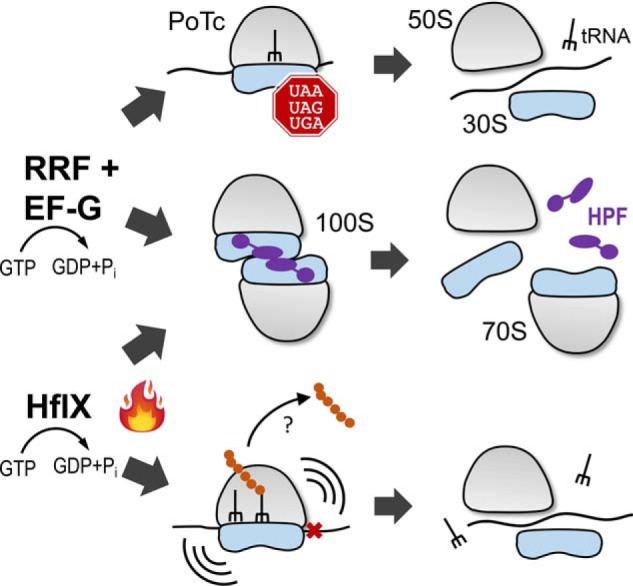
**Disassembly pathways of *S. aureus* 100S ribosomes.** RRF and EF-G act in concert to split the PoTc in a GTP hydrolysis-dependent manner. The hibernating 100S ribosome is the second substrate of RRF/EF-G. Thermal stress induces the expression of *hflX*. HflX recycles 100S ribosomes and rescues stalled 70S ribosomes. A *question mark* indicates an unknown factor responsible for the hydrolysis of peptidyl-tRNA. HPF is colored in *purple*.

In addition to the HflX-dependent disassembly pathway (31), we report here that the 100S ribosome is a previously unrecognized target of RRF and EF-G in *S. aureus*. RRF and EF-G split the 100S complexes via EF-G-mediated GTP hydrolysis, similar to the GTPase activity of HflX (Fig. 1). The two pathways appear to operate under different conditions, with RRF/EF-G acting as the primary dissociation factor for 100S dimers, whereas HflX serves as a secondary factor that is tightly regulated by heat stress. In fact, RRF and EF-G are highly abundant (∼20 μm) during active growth (46, 51), whereas HflX expression is almost undetectable at this stage (31). In addition, RRF/EF-G and HflX bind to the overlapping regions of the ribosome (32), further suggesting that the dissociation events mediated by these two pathways are mutually exclusive. When HflX is overexpressed in a plasmid, it functionally complements the loss of RRF that acts to recruit EF-G (Fig. 4). RRF/EF-G and HflX also recycle ribosomes from PoTc and thermally arrested ribosomes, respectively (Fig. 6) (32). It is conceivable that the existence of these distinct yet overlapping pathways is evolutionarily favorable for cells to cope with various cellular stresses.

Structural and biochemical studies revealed that RRF/EF-G and HflX bind to the 50S subunit and occupy the 30S–50S intersubunit space, presumably disrupting the intersubunit bridge B2a between helix 44 and helix 69 via GTP hydrolysis (32, 33, 38, 52). The sequence of events in 100S ribosome disassembly remains unclear. It is possible that the dimers are first split into 70S monomers, followed by dissociation into 30S and 50S subunits. The dimers may concurrently dissociate into 30S and 50S subunits in a single step. Alternatively, their dissociation may involve both single-step and two-step events. It has been shown that subunit dissociation is not essential for translational reinitiation. Instead, initiation could occur in a 70S-scanning mode (53, 54), and cells carrying a covalently linked 70S ribosomes are viable (55). Future kinetic studies are needed to dissect the precise order of 100S disassembly and the departure of HPF and the dissociation factors from the ribosomes. Furthermore, RRF depletion evokes ribosome queuing and translational read-through (Fig. 5). In principle, unrecycled ribosomes could allow reinitiation at the downstream cotranscribed ORF or reinitiation at the first AUG-like codon, provided that fMet-tRNA^fMet^ is available. Alternatively, ribosome queuing could trigger one of the ribosome rescue systems (56, 57) that target stalled elongating ribosomes and the terminating ribosomes. Previous studies have employed heterologous reporter mRNAs to investigate 70S-scanning reinitiation (53, 54), the global impact of RRF insufficiency is not well-understood. Ribosome profiling and MS can be performed to address this outstanding question but are currently beyond the scope of this study.

The *in vivo* and *in vitro* activity of the temperature-sensitive *E. coli* RRF(V117D) mutant (equivalent to *S. aureus* V116D) has been extensively studied (45, 58, 59). At a nonpermissive temperature, *E. coli* RRF(V117D) cells are nonviable because the protein is misfolded and rapidly degraded at high temperature. Surprisingly, we found that the recombinant *S. aureus* RRF(V116D) protein is fully active, and perhaps more potent in dissociating 100S ribosomes *in vitro* (Fig. 2*B*). The overexpression of RRF(V116D) in a multicopy plasmid overcomes the growth defects of the RRF-depleted strain (Fig. 3). These observations are supported by findings that the RRF(V116D) protein is fully competent in ribosome binding and EF-G recruitment (Figs. S1 and S2) and that an excess amount of RRF(V116D) can compensate for the instability of the mutant protein *in vivo*. Consistent with this notion, overexpression of *E. coli* RRF(V117D) eliminates the temperature-sensitive phenotype (45).

The phenotype of the RRF-depleted strain is more severe on solid media than in the liquid media (Fig. 3, *C* and *D*). These striking differences could be due to the local depletion of nutrients and moisture or the accumulation of a toxic compound on the agar plates. For instance, specific solidifying agents may sequester essential micronutrients or contain toxic ingredients. Growth defects caused by the chelation of zinc on the agar plates have been observed in a zinc transporter mutant (60). To test this possibility, we compared the growth defects of the RRF-depleted strain on plates containing routinely used Bacto^TM^ agar, Gelrite that was free of phenolic compounds, agarose with a low ash content, and Eiken agar that retained more water. Although agarose and Eiken agar supported increased growth, the defects remained severe (Fig. S3), ruling out the contribution of a solidifying agent. It is possible that shaken liquid culture may provide additional aeration and spatial accessibility to nutrients, resulting in better growth.

Ribosome availability is directly linked to bacterial growth and revival, and ribosome homeostasis is essential for cellular function. The identification of the dual action of RRF/EF-G in maintaining a normal translational cycle and reactivating dormant ribosomes implies that RRF is an ideal antimicrobial target for inhibiting both the proliferation and regrowth of pathogens.

## Experimental procedures

### Bacterial strains, plasmids, and growth conditions

The strains and plasmids used in this study are listed in Table S1. The methicillin-resistant *S. aureus* (MRSA) USA300 strain JE2 (GenBank CP000255) was used throughout the study. The deletion mutant of *hflX* (SAUSA300_1198) has been described previously (31). Unless otherwise noted, *S. aureus* cells were grown at 37 °C in tryptic soy broth (TSB, Difco) at a 5:1 tube- or flask-to-medium ratio with a 1:100 dilution of an overnight seed culture. TSB agar plates were prepared using Difco^TM^ agar (Difco, BD 281230), Eiken agar (Eiken, E-MJ00), Gelrite (RPI, G35020), and agarose (GenMate, E-3120). When necessary, erythromycin, chloramphenicol, cadmium chloride, kanamycin, and anhydrotetracycline (all from Sigma-Aldrich) were used at 5 μg/ml, 10 μg/ml, 0.15 mm, 75 μg/ml, and 400 ng/ml, respectively. The restriction-deficient *S. aureus* RN4220 strain was used as a plasmid passage surrogate before the plasmid was transformed into the destination JE2 derivatives. *E. coli* cells harboring expression vectors were grown at 16 or 37 °C in LB (Difco). Antibiotics were used at 50 (kanamycin) or 100 μg/ml (ampicillin, Sigma-Aldrich). All GTP analogs were from Sigma-Aldrich. Primers were purchased from IDT DNA and are listed in Table S2.

#### 

##### Construction of conditional RRF knockdown strains MNY162 and MN165

An ∼0.7-kb DNA containing N terminally His_6_-tagged *frr* (SAUSA300_1152) was PCR amplified from *S. aureus* JE2 genomic DNA using the primers P1209 and P1210. The fragment was cloned into the KpnI and SacI sites of pRMC2 (61), generating pRMC2His_6_RRF under the control of the Tet promoter. The P_Tet_His_6_-*frr* region was released from pRMC2His_6_RRF by PstI and SacI digestion and cloned into the same sites of pJC1111 (48), generating pJC1111-P_Tet_His_6_-*frr*. This suicide plasmid was integrated into the chromosome of strain RN9011 following standard protocols (48). The P_Tet_His_6_-*frr* allele was subsequently transferred to the JE2 strain via Φ11 phage transduction, resulting in MNY147 bearing the native *frr* and an extra copy of P_Tet_His_6_-*frr*. The WT *frr* allele was deleted from MNY147 by homologous recombination using the plasmid pBT2Δfrr::Km. Two independent allelic exchanges were performed, generating the RRF-depleted strains MNY162 and MN165. To construct the pBT2Δfrr::Km plasmid, 1-kb flanking regions of *frr* were amplified with the primer pairs P1231/P1232 and P1233/P1234 using a two-step PCR protocol with JE2 genomic DNA as the template. The 2-kb SmaI-containing fragment was digested with SacI and SalI and cloned into the same sites of pBT2 (62) to produce pBT2Δfrr. A 1.4-kb kanamycin resistance cassette was released from pBTK (63), end repaired, and cloned into the SmaI site of pBT2Δfrr, generating pBT2Δfrr::Km.

##### Complementation plasmids

pLI50 (64) was used for genetic complementation. The construction of phflX was reported previously (31). To construct the *frr* complementation plasmid, a constitutively expressed CodY-dependent promoter (28) was attached to the *frr* via two-step PCR using the primer pairs P651/P1244 and P1245/P1121 with JE genomic DNA as the template. The PCR product was digested with BamHI and EcoRI and cloned into the same sites in pLI50, generating the pfrr plasmid. To construct pfrr(V116D), a site-directed QuikChange mutagenesis kit (Agilent Genomics) was employed to introduce a V116D substitution using the primers P1219/P1220.

##### Overexpression and reporter plasmids

The pET28a vector was used to overexpress HflX, RRF, and EF-G recombinant proteins. pET28-HflX has been described previously (31). To construct N terminally His_6_-tagged RRF, the *frr* coding region was PCR amplified with P1120/P1121 primers using JE2 genomic DNA as the template. The product was cloned into the NdeI and EcoRI sites of pET28a. The A28E and V116D alleles were introduced by QuikChange mutagenesis using primer pairs P1217/P118 and P1219/P1220, respectively. To construct C terminally His_8_-tagged EF-G, *fusA* (locus SAUSA300_0532) was PCR amplified with P1167/P1168, and the fragment was cloned into the NcoI and XhoI sites of pET28a. The R29A, R59A, and H572A mutations were introduced into EF-G by QuikChange mutagenesis using primer pairs P1121/P1122, P1213/P1214, and P1215/P1216, respectively. The K54stop mutation was introduced to the *gfp* reporter in pDM4 by QuikChange mutagenesis using primers P1255/P1256.

### Native 100S ribosome isolation and dissociation

Crude ribosomes were isolated from late log-phase JE2 strains by cryo-milling methods (4, 25). Fifty absorbance units (*A*_260_) of ribosomes were layered on a 5–25% sucrose gradient that was prepared on a BioComp Gradient Master. The samples were centrifuged at 210,000 × *g* at 4 °C in a SW41 rotor in a Beckman Coulter Optima XPN-100 ultracentrifuge for 3 h. Fractionation was performed using a Brandel fractionation system equipped with a UA-6 UV detector. The 100S ribosome peaks were collected and centrifuged in a SW55 Ti rotor at 246,000 × *g* at 4 °C for 18 h. The ribosome pellet was resuspended in low-salt buffer (20 mm Hepes, pH 7.5, 14 mm Mg(OAc)_2_, 100 mm KOAc, 0.5 mm phenylmethylsulfonyl fluoride, 1 mm DTT) and quantified according to the *A*_260_ value (1 *A*_260_ = 23 pmol/ml of 70S).

*In vitro* 100S dissociation samples were assembled with a 0.2 μm 100S ribosomes, 2 μm proteins (RRF, EF-G, or HflX), and 2 mm GTP analogs in a 100-μl reaction. The reactions were incubated at 37 °C for 30 min before being spun through a 5–20% sucrose gradient. Ribosome profiles were recorded in real time traces probed at an absorbance of 254 nm. For quantification of 100S to 70S ratios, the baseline was first defined as the global minimum excluding the free nucleic acids and protein peak. The boundary of 100S–70S were manually selected from the trough between the peaks. The area under a peak was calculated by ImageJ and divided to obtain the ratio.

For ribosome queuing analysis, cells were grown in TSB at 37 °C until *A*_600_ ∼ 1.4 and harvested by rapid filtration and freezing in liquid nitrogen without the addition of chloramphenicol typically used to arrest translation in the ribosome profiling experiments (65). Frozen pellets were cryogenically pulverized on a Retsch Miller as described previously (4, 25). Four *A*_260_ units of crude ribosomes were centrifuged through a 10–40% sucrose gradient to separate the ribosome particles. To collapse polysomes, crude ribosomes were untreated or treated with increasing concentrations (5–20 μg/ml) of RNase A (Ambion AM2271) at 25 °C for 1 h prior to ultracentrifugation.

### Purification of recombinant proteins and antibody production

The overexpression and purification of the His-tagged recombinant proteins have been described in detail previously (4, 31) and some minor modifications were made in protocol for bacterial growth for EF-G production. The overexpression of EF-G at 37 °C results in an insoluble protein. Cells carrying the EF-G plasmid were grown at 37 °C until an *A*_600_ of ∼0.4, after which isopropyl 1-thio-β-d-galactopyranoside was added at a final concentration of 0.5 mm, and induction was performed at 16 °C for 16 h before harvest. Polyclonal antibodies were raised in rabbits by Josman, LLC.

### Western blotting

Total *S. aureus* total lysates were prepared in a bead beater as described previously (28). Immunoblotting was performed as reported (28, 66). Antibodies were used at the following dilution rates: anti-RRF (1:4,000), anti-HflX (1:1,000 dilutions after pre-absorption with lysates of Δ*hflX* knockout), and anti-EF-G (1:4,000).

### GTPase activity assay

The P_i_ColorLock Gold kit (Innova Biosciences) was used to determine the GTP hydrolysis of EF-G variants by measuring the amount of free P_i_. Detailed protocols were reported previously (31).

### GFP reporter assays

*S. aureus* cells harboring the pDM4 or pDM4(K54stop) plasmid were grown at 37 °C in TSB in the presence or absence of 400 ng/ml of aTc. Cells were harvested at an *A*_600_ of ∼0.3 by centrifugation. The pellets were resuspended and washed three times with sterile 1× PBS buffer to remove background fluorescence from TSB medium. The cell density was adjusted to an *A*_600_ of ∼0.2. Two-hundred microliters of the cell suspension was transferred to a black 96-well–flat-bottom plate (Corning Costar®) in triplicate, and GFP fluorescence was measured on a TECAN SPARK plate reader at excitation and emission wavelengths of 485 and 535 nm, respectively. The final fluorescence units were calculated by subtracting the fluorescence from the JE2 background and dividing the results by the final *A*_600_ to correct for cell density. The experiments were repeated five times. Statistical significance was evaluated with unpaired *t* test.

### Data availability

All data are contained within the manuscript

## Author contributions

A. B. data curation; A. B. and M.-N. Y. formal analysis; A. B. validation; A. B., K. E. S., and M.-N. Y. investigation; A. B. visualization; A. B., K. E. S., and M.-N. Y. methodology; A. B. and M.-N. Y. writing-original draft; M.-N. Y. conceptualization; M.-N. Y. resources; M.-N. Y. supervision; M.-N. Y. funding acquisition; M.-N. Y. project administration; M.-N. Y. writing-review and editing.

## Supplementary Material

Supporting Information

## Footnotes

2 The abbreviations used are:

HPF: hibernation-promoting factor
RMF: ribosome modulation factor
RRF: ribosome-recycling factor
PoTc: post-termination complex
EF-G: GTPase elongation factor-G
aTc: anhydrotetracyline
GMPPNP: guanosine 5′-(β,γ-imido)triphosphate
TSB: tryptic soy broth.
