# Supplementary material for: The hibernating 100S complex is a target of ribosome-recycling factor and elongation factor G in Staphylococcus aureus
Source: J Biol Chem. 2020 Mar 24;295(18):6053–63. doi: 10.1074/jbc.RA119.012307 (PMC7196661; doi:10.1074/jbc.RA119.012307)
Supplement: Supporting Information [file supp_295_18_6053__index.html]

The hibernating 100S complex is a target of ribosome-recycling factor and elongation factor G in Staphylococcus aureus — Recycling of S. aureus 100S ribosomes — The hibernating 100S complex is a target of ribosome-recycling factor and elongation factor G in Staphylococcus aureus — Recycling of S. aureus 100S ribosomes — Supporting Information 

# The hibernating 100S complex is a target of ribosome-recycling factor and elongation factor G in *Staphylococcus aureus*

## Supporting Information

- Supporting Information (to be published online) - Supporting table and figures
