# Supplementary material for: The hibernating 100S complex is a target of ribosome-recycling factor and elongation factor G in Staphylococcus aureus
Source: J Biol Chem. 2020 Mar 24;295(18):6053–63. doi: 10.1074/jbc.RA119.012307 (PMC7196661; doi:10.1074/jbc.RA119.012307)
Supplement: Supporting Information [file supp_RA119.012307_157704_2_supp_495450_q7pyws.pdf]

Supporting Information

The hibernating 100S complex is a target of ribosome recycling factor and elongation factor G in *Staphylococcus aureus*

Arnab Basu[1], Kathryn E. Shields[1], and Mee-Ngan F. Yap[1,2*]

From the [1]Department of Biochemistry and Molecular Biology, Saint Louis University School of Medicine, Saint Louis, MO 63104; [2]Department of Microbiology-Immunology, Northwestern University Feinberg School of Medicine, Chicago, IL 60611.

Running title: *Recycling of S. aureus 100S ribosomes*

[*]To whom correspondence should be addressed: Mee-Ngan F. Yap, Department of Microbiology-Immunology, Northwestern University Feinberg School of Medicine, Chicago, IL 60611; frances.yap@northwestern.edu; Tel. (312) 503-3793

1) **Table S1.** Strains and Plasmids.

2) **Table S2.** Oligonucleotide primers.

3) **Figure S1.** EF-G (R59A), RRF(A28E) and RRF(V116D) mutations do not affect ribosome interactions.

4) **Figure S2.** Analysis of RRF mutant proteins.

5) **Figure S3.** Effects of solidifying agents on the growth of the RRF-depleted strain.

6) **Figure S4.** HflX is exclusively expressed during heat stress.

7) **Supporting Information References**

Table S1. Strains and plasmids.

Name	Genotypes and relevant features	Source
S. aureus		
JE2	Parental strain, plasmid cured LAC strain	BEI resources NR-46543
RN4220	*sau1⁻, hsdR⁻, mec⁻, rsbU⁻, agr⁻*	ATCC NR-45946
RN9011	RN4220/pRN7023 (SaPI1 integrase), Cm^R	(1)
MNY102	Δ*hflX*::Erm	(2)
MNY147	*frr*⁺, P_{Tet}-*frr*, $CdCl_2^R$	This work
MNY162[a]	Δ*frr::Km, frr*⁺, P_{Tet}-*frr*; $CdCl_2^R$, Km^R	This work
MNY165[a]	Δ*frr::Km, frr*⁺, P_{Tet}-*frr*; $CdCl_2^R$, Km^R	This work
E. coli		
BL21(DE3)	F⁻ *ompT gal dcm lon hsdS$_B$*($r_B^-m_B^-$) λ(DE3 [*lacI lacUV5-T7p07 ind1 sam7 nin5*]) [*malB*⁺]$_{K\text{-}12}$($λ^S$)	Lucigen
**Plasmids**		
pRMC2	*E. coli-S. aureus* shuttle vector, Tet-inducible promoter, Amp^R, Cm^R	(3)
pLI50	*E. coli-S. aureus* shuttle vector, Amp^R, Cm^R	(4)
pBT2	Temperature sensitive, *E. coli-S. aureus* shuttle vector, Amp^R, Cm^R	(5)
pBTK	Temperature sensitive, *E. coli-S. aureus* shuttle vector, 1.4-kb *aph-A3* cloned into *SmaI* site of pBT2, Amp^R, Cm^R, Km^R	(6)
pJC1111	Suicide vector, Amp^R, $CdCl_2^R$	(1)
p*hflX*	Native promoter driven-*hflX* on pLI50, Amp^R, Cm^R	(2)
pDM4	*gfp* under control of a P_{cidABC} promoter, Amp^R, Cm^R	(7)
pDM4(K54stop)	*gfp(K54stop)* under control of a P_{cidABC} promoter, Amp^R, Cm^R	This work
p*frr*	*frr* on pLI50 under control of a *codY*-dependent promoter, Amp^R, Cm^R	This work
p*frr*(V116D)	*frr*(V116D) on pLI50 under control of a *codY*-dependent promoter, Amp^R, Cm^R	This work
pJC1111-P_{Tet}His$_6$-*frr*	P_{Tet}His$_6$-*frr* on pJC111, Amp^R, $CdCl_2^R$	This work
pBT2Δ*frr*::Km	Δ*frr*::Km allele on pBT2, ts, Amp^R, Cm^R	This work
pET28a	Overexpression plasmid, Km^R	Novagen
pET28_HflX	*hflX* on pET28a, Km^R; N-terminally 6His-tagged HflX	(2)
pET28_RFF	*frr* on pET28a, Km^R; N-terminally 6His-tagged RRF	This work
pET28_RFF (A28E)	*frr(A28E)* on pET28a, Km^R; N-terminally 6His-tagged RRF(A28E)	This work
pET28_RFF (V116D)	*frr(V116D)* on pET28a, Km^R; N-terminally 6His-tagged RRF(V116D)	This work
pET28_EF-G	*fusA* on pET28a, Km^R; C-terminally 8His-tagged EF-G	This work
pET28_EF-G (R29A)	*fusA(R29A)* on pET28a, Km^R; C-terminally 8His-tagged EF-G (R29A)	This work
pET28_EF-G (R59A)	*fusA(R59A)* on pET28a, Km^R; C-terminally 8His-tagged EF-G (R59A)	This work
pET28_EF-G (H572K)	*fusA(H572K)* on pET28a, Km^R; C-terminally 8His-tagged EF-G (H572K)	This work

[a] independent clones of RRF-depleted strains.

Table S2. Oligonucleotide primers.

Name	Number	Sequence (5' →3')
R29A EF-G(F)	P1121	ACGACTACGACTGAAGCTATTCTTTATTACACT
R29A EF-G(R)	P1212	AGTGTAATAAAGAATAGCTTCAGTCGTAGTCGT
R59A EF-G(F)	P1213	GAGCAAGAACAAGACGCTGGTATTACTATCACA
R59A EF-G(R)	P1214	TGTGATAGTAATACCAGCGTCTTGTTCTTGCTC
H572K EF-G(F)	P1215	TATGATGGTTCATACAAAGATGTCGATTCATCT
H572K EF-G(R)	P1216	AGATGAATCGACATCTTTGTATGAACCATCATA
A28E RRF(F)	P1217	TTAGCTAACATCAGTGAAGGAAGAGCTAATTCA
A28E RRF(R)	P1218	TGAATTAGCTCTTCCTTCACTGATGTTAGCTAA
V116D RRF(F)	P1219	GAGCGCGTTAAAGATGATAAGAAAATTGGTGAA
V116D RRF(R)	P1220	TTCACCAATTTTCTTATCATCTTTAACGCGCTC
sGFP K54 stop (F)	P1255	TTTGCACTACTGGATAACTACCTGTTCCG
sGFP K54 stop (R)	P1256	CGGAACAGGTAGTTATCCAGTAGTGCAAA
pET28a (F)	P457	TAATACGACTCACTATAGGG
pET28a (R)	P458	GCTAGTTATTGCTCAGCGG
EF-G(F)	P1167	CGCCATGGCTAGAGAATTTTCATTAGAA
EF-G_His(R)	P1168	GCCCTCGAGTTAGTGGTGGTGGTGGTGGTGTTCACCTTTAT TTTTCTTGATAAT
6HisRRF(KpnI)f	P1209	TAGGTACCAAATAAATTTAGAGGTGTAAAATAATGCATCA TCACCATCATCATCACGGTAGTGGAAGTGACATTATTAAT GAAACTAAATCAAG
RRF(SacI)r	P1210	TAAGAGCTCATTGTATATTAGTTTTATACTGACATAATATC
Safrr1_SacI_f	P1231	GTTGAGCTCGTACGCTATGAAGTAGGCGAAG
Safrr2_SmaI_r	P1232	TGCCGCGTACTCTGCGCCCGGGATACTCATTATTTTACA CCTCTAAATTTATTTTGT
Safrr3_SmaI_f	P1233	ATCCCGGGCGCAGAGTACGCGGCATAAAACTAATATA CAATGACATATTAAAATG
Safrr4_SalI_r	P1234	TCAGTCGACGAAACTTGCCAAATCAAGAAATTACT
SaHPFp(BamHI)f	P651	CGGGATCCATACAACTGGATTAACAATTCATCGTG CAGGGTG
P_{hpf}-frr_R	P1244	AGTTTCATTAATAATGTCACTCATAGTAATCTCTCCTT AAACCTCTTTAT
P_{hpf}-frr_F	P1245	ATAAAGAGGTTTAAGGAGAGATTACTATGAGTGACA TTATTAATGAAACT
SaRRF(EcoRI)r	P1121	CATTGTGAATTCGTTTTATA TGACATAATATCTTTTTC
SaRRF(NdeI)f	P1120	GTAAACATATGAGTGACATTATTAATGAAAC

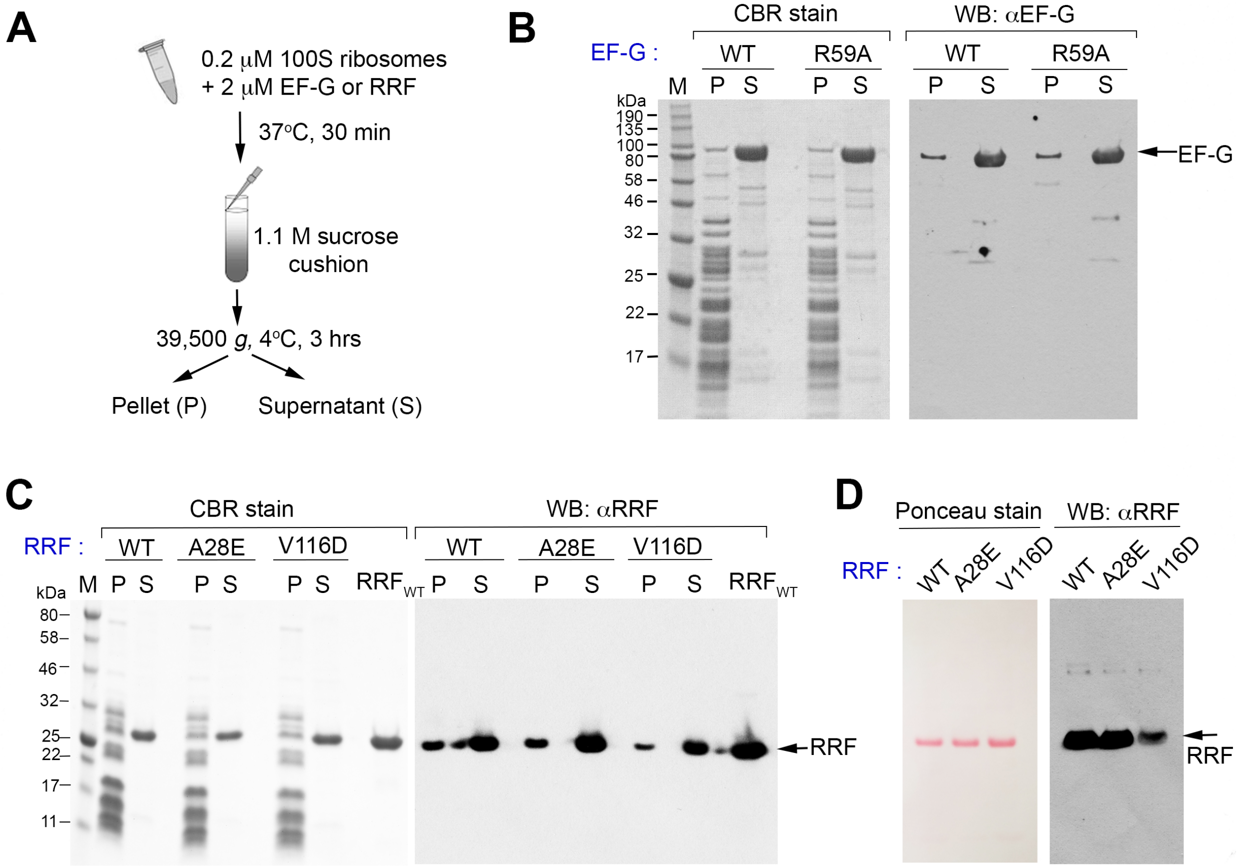

Figure S1. EF-G (R59A), RRF(A28E) and RRF(V116D) mutations do not affect ribosome interactions. (A) Experimental scheme of the ribosome-binding assay. RRF-bound or EF-G-bound ribosomes are co-sedimented in the pellet (P) fraction after ultracentrifugation. Free forms of RRF and EF-G are present in the supernatant (S). **(B)** EF-G(R59A) binds to the ribosome in a comparable manner to WT EF-G. Immunoblotting of co-sedimented fractions against an anti-EF-G antibody (1:4,000 dilutions). **(C)** The ribosome binding of RRF mutants is comparable to the WT RRF. As in panel (B), Western blotting (1:4,000 dilutions of anti-RRF) was used to assess the amount of ribosome-bound RRF. The weaker signals of the V116D mutant are not due to impaired ribosome binding but to the poor reactivity of the V116D mutant protein to the antibody (see panel D). Recombinantly purified RRF(RRF_{WT}) serves as the marker. M, protein marker. **(D)** Anti-RRF does not recognize the V116D mutant as efficiently as the WT or its A28E mutant in a Western blot. Equal amounts of purified recombinant RRF proteins were loaded onto SDS-PAGE gels and probed with anti-RRF (1:4,000 dilutions).

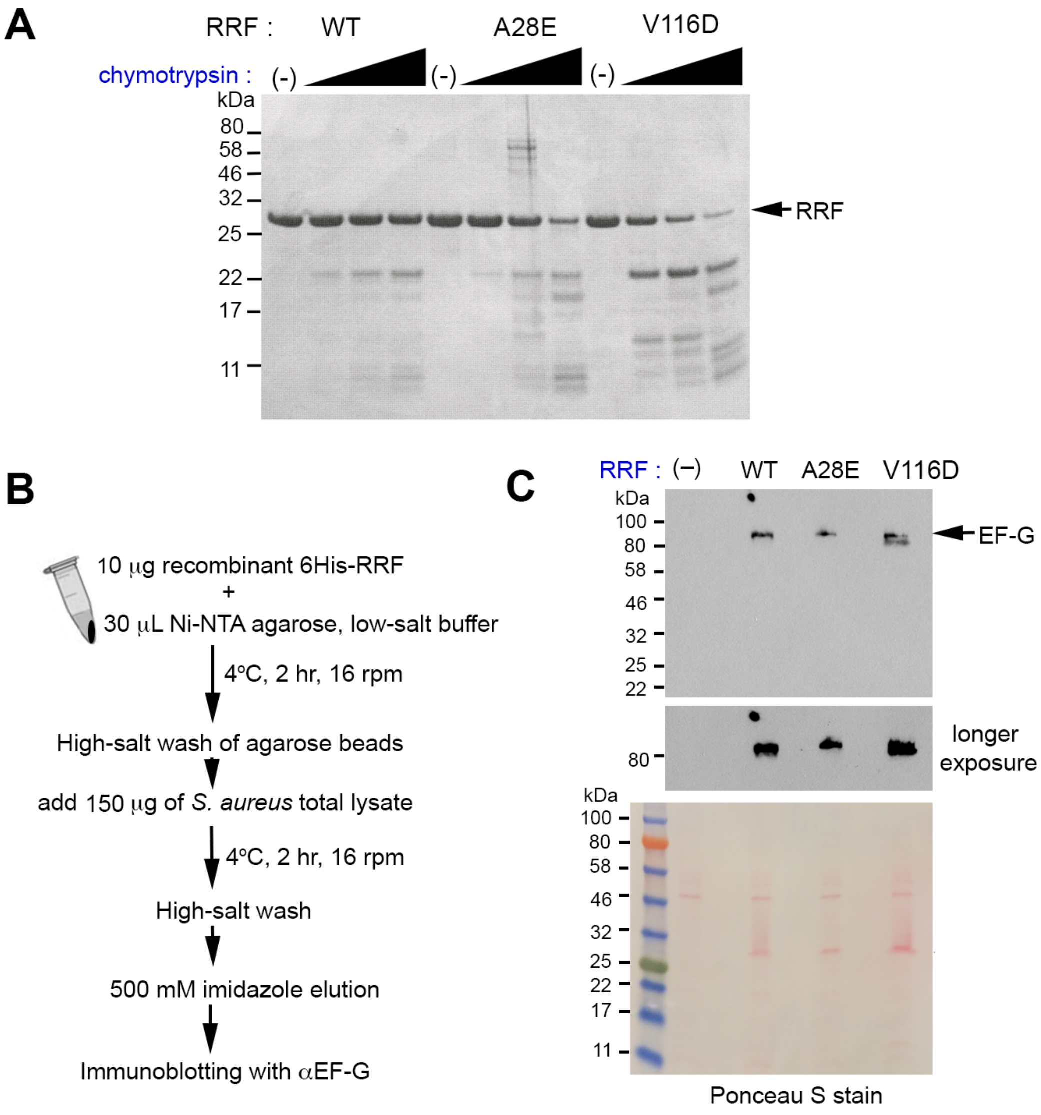

Figure S2. Analysis of RRF mutant proteins. (A) Limited proteolysis with chymotrypsin revealed significant conformational changes in the RRF(V116D) mutant protein. RRFs at 2 μM were incubated with immobilized chymotrypsin (Princeton Separations) at increasing concentrations (0, 0.002 mU, 0.01 mU, 0.2 mU). The reactions were performed at 37°C for 20 min and stopped by the addition of 1 mM phenylmethylsulfonyl fluoride (PMSF). The samples were precipitated with acetone and analyzed in a 4-12% Bis-Tris/MES SDS-PAGE gel. The V116D mutant shows much greater proteolytic susceptibility compared to the WT and the A28E mutant. **(B)** Experimental procedures of the affinity pull-down assay presented in panel C. **(C)** The RRF mutants capture cellular EF-G as effectively as the WT RRF. The recombinant His$_6$-tag proteins of the RRF mutants were used as the bait to pull down EF-G from the total cell lysates, followed by detection via Western blotting using anti-EF-G (1:4,000 dilutions).

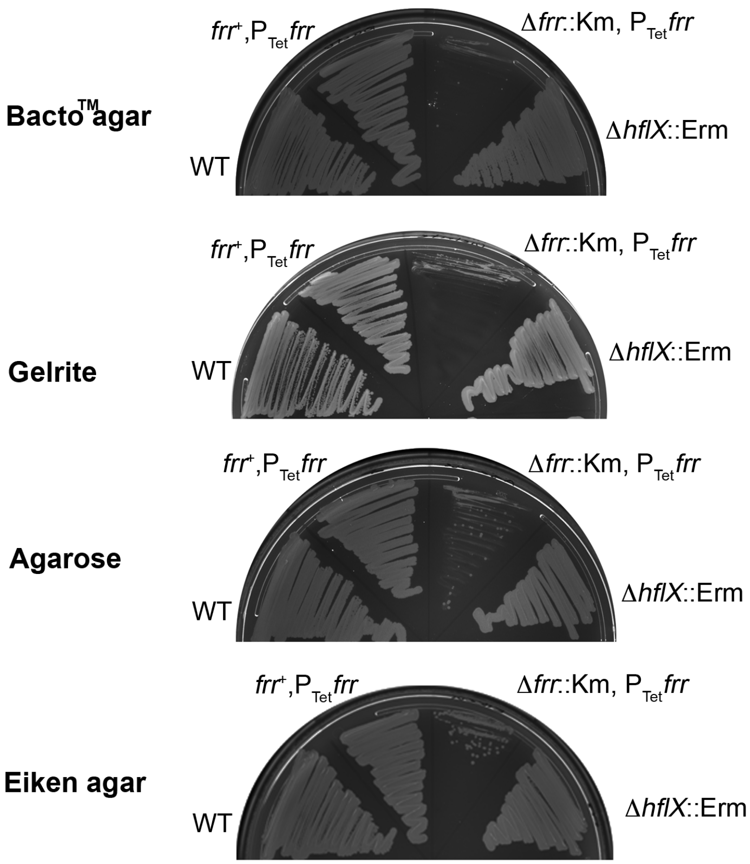

Figure S3. Effects of solidifying agents on the growth of the RRF-depleted strain. Agarose with a lower ash content and Eiken agar holding more moisture support better growth of the RRF-depleted strain (Δ*frr*::Km,P_{Tet}-*frr*) in the absence of the anhydrotetracycline (aTc) inducer. Tryptic soy broth (TSB)-based agar plates were prepared with conventional Bacto™ agar (same as in Figs. 4-5), the phenolic compound-free Gelrite, agarose or Eiken agar without aTc supplementation.

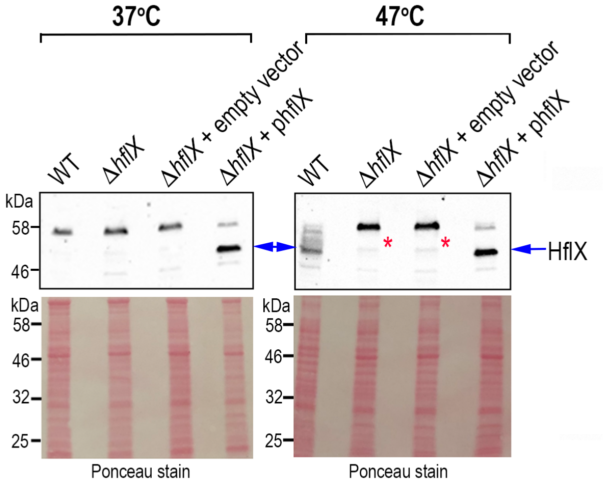

Figure S4. HflX is exclusively expressed during heat stress. Lysates were extracted from logarithmically growing *S. aureus* tryptic soy broth (TSB) cultures at 37°C, or TSB cultures after 2 hr of heat shock at 47°C. Total protein (125 µg/lane) was resolved by SDS-PAGE and probed with anti-HflX (1:1,000 dilutions) that were preabsorbed with lysates from the Δ*hflX* knockout to reduce non-specific cross-reaction. The plasmid-borne *hflX* overexpressing strain served as a positive control. HflX was undetectable at 37°C. Blue arrows indicate HflX bands. A red asterisk marks the nonspecific band migrates slightly slower than HflX.

Supporting information references

1. Chen, J., Yoong, P., Ram, G., Torres, V. J., and Novick, R. P. (2014) Single-copy vectors for integration at the SaPI1 attachment site for *Staphylococcus aureus*. *Plasmid* **76**, 1-7
2. Basu, A., and Yap, M. N. (2017) Disassembly of the *Staphylococcus aureus* hibernating 100S ribosome by an evolutionarily conserved GTPase. *Proc Natl Acad Sci U S A* **114**, E8165-E8173. PMCID: PMC5625922
3. Corrigan, R. M., and Foster, T. J. (2009) An improved tetracycline-inducible expression vector for *Staphylococcus aureus*. *Plasmid* **61**, 126-129
4. Lee, C. Y., Buranen, S. L., and Ye, Z. H. (1991) Construction of single-copy integration vectors for *Staphylococcus aureus*. *Gene* **103**, 101-105
5. Bruckner, R. (1992) A series of shuttle vectors for *Bacillus subtilis* and *Escherichia coli*. *Gene* **122**, 187-192
6. Fuller, J. R., Vitko, N. P., Perkowski, E. F., Scott, E., Khatri, D., Spontak, J. S., Thurlow, L. R., and Richardson, A. R. (2011) Identification of a lactate-quinone oxidoreductase in *Staphylococcus aureus* that is essential for virulence. *Front Cell Infect Microbiol* **1**, 19
7. Moormeier, D. E., Endres, J. L., Mann, E. E., Sadykov, M. R., Horswill, A. R., Rice, K. C., Fey, P. D., and Bayles, K. W. (2013) Use of microfluidic technology to analyze gene expression during *Staphylococcus aureus* biofilm formation reveals distinct physiological niches. *Appl Environ Microbiol* **79**, 3413-3424
